# Mesoporous TiO_2_ coating on carbon–sulfur cathode for high capacity Li–sulfur battery†

**DOI:** 10.1039/c8ra01380b

**Published:** 2018-03-26

**Authors:** Ruchira Dharmasena, Arjun Kumar Thapa, Ram Krishna Hona, Jacek Jasinski, Mahendra K. Sunkara, Gamini U. Sumanasekera

**Affiliations:** Conn Center for Renewable Energy Research, University of Louisville KY USA mahendra@louisville.edu; Department of Physics & Astronomy, University of Louisville KY USA gamini.sumanasekera@louisville.edu; Department of Chemistry, University of Louisville KY USA

## Abstract

In this paper, a meso-porous TiO_2_ (titania) coating is shown to effectively protect a carbon–sulfur composite cathode from polysulfide dissolution. The cathode consisted of a sulfur impregnated carbon support coated with a few microns thick mesoporous titania layer. The carbon–sulfur cathode is made using activated carbon powder (ACP) derived from biomass. The mesoporous titania coated carbon–sulfur cathodes exhibit a retention capacity after 100 cycles at C/3 rate (433 mA g ^−1^) and stabilized at a capacity around 980 mA h g^−1^. The electrochemical impedance spectroscopy (EIS) of the sulfur cathodes suggests that the charge transfer resistance at the anode, (*R*_act_) is stable for the titania coated sulfur electrode in comparison to a continuous increase in *R*_act_ for the uncoated electrode implying mitigation of polysulfide shuttling for the protected cathode. Stability in the cyclic voltammetry (CV) data for the first 5 cycles further confirms the polysulfide containment in the titania coated cathode while the uncoated sulfur electrode shows significant irreversibility in the CV with considerable shifting of the voltage peak positions. Raman spectroscopy and X-ray photoelectron spectroscopy (XPS) studies confirm the adsorption of soluble polysulfides by mesoporous titania.

## Abstract 2

Mesoporous TiO_2_ coating on carbon–sulfur cathode with simple electrical contact for high capacity Li–S battery.

## Graphical abstract

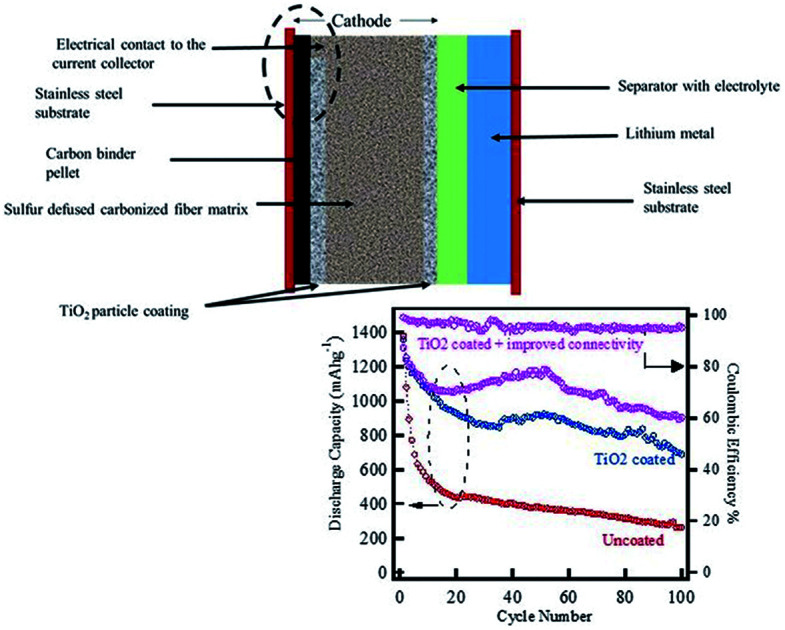

## Introduction

Lithium–air and lithium–sulfur batteries have attracted worldwide attention due to their potential for achieving higher energy density than lithium ion battery technology.^1^ Sulfur as a cathode has an excellent theoretical gravimetric discharge capacity of 1672 mA h g^−1^ and Li–air has a theoretical discharge capacity of ∼3623 W h kg^−1^ corresponding to Li_2_O_2_ (3840 mA h g^−1^ excluding O_2_ mass).^1,2^ Li–air batteries require pure O_2_ at the cathode due to side reactions caused by the moisture and CO_2_ present in air. These can lead to a range of issues such as cathode instabilities, catalyst driven side reactions, electrolyte instabilities and the reactivity of Li_2_O_2_ and its intermediates. The majority of recent Li–O_2_ reports have only assessed battery performance at limited depths of discharge using pure O_2_ and impractical low mass loadings, and this makes it difficult to gauge their prospects in practical applications.^3^

On the other hand, Li–S batteries seem closer to industrial readiness if poor cyclability is addressed through proper sulfur cathode formulations.^4^ The poor cyclability of Li–S batteries is attributed to two main factors: poor electrical conductivity of sulfur and dissolution of polysulfides into the electrolyte during lithiation and delithiation. The lithiation of sulfur is facilitated by the formation of polysulfide intermediates. Electrochemically, elemental sulfur first reduces to S_8_^2−^ and forms Li_2_S_8_ in liquid form. Li_2_S_6_ and Li_2_S_4_ are formed thereafter.^5,6^ As a result of polysulfide dissolution, the phenomena known as polysulfide shuttle will occur causing active material inaccessible for further electrochemical reactions.^7,8^ Typically, polysulfides Li_2_S_*n*_ (2 < *n* < 8) are known to dissolve in organic electrolytes. Polysulfide shuttle phenomenon has been studied extensively.^7^ Among organic solvents, 1,2-dimethoxyethane (DME) and 1,3-dioxolane (DOL) based organic solvents are preferable due to their bulky anions which can effectively reduce polysulfide solubility.^9^ According to Barchasz *et al.*^10^ dissolution of polysulfide is also a necessity for the proper operation of the sulfur electrode. It is also claimed that dissolution of polysulfide increases the viscosity of the electrolyte and hence less viscous solvents are preferable for Li–S batteries. There are three ways to improve the cyclability and durability of sulfur cathode: first, the sulfur particles need to be encapsulated to minimize the leaking of polysulfides, second, the electrode material must be properly wetted by electrolyte, and lastly a good electronic conductivity must be maintained within the bulk electrode. In addition, there are some reports that indicate gas evolution in Li–S cells could also be problematic for their practical applications.^11^

In the last few years, many techniques have been attempted to prevent dissolution of polysulfides from sulfur cathodes into the electrolyte. An excellent review of these techniques can be found in ref. 12. Use of meso/micro pore carbon structures has been attempted to trap soluble polysulfides. A method using micro pore structure was used to limit the chain length of resulting polysulfides during lithiation and avoid long chain soluble polysulfides.^13^ The use of microporous carbon material has been found to yield high initial discharge capacity but resulted in continuous decay.^1^ In another concept, a meso/micro ordered carbon architecture–sulfur composite is synthesized by first creating the carbon structure using a silicon template followed with diffusion of sulfur.^14^ The advantage of such meso-porosity for a sulfur cathode is that electrolyte can channel into the micro-pore sites. In another technique, sulfur nano-particles were encapsulated by carbonized polymer coating.^15^ Such encapsulated sulfur particles tend to expand when polysulfides are formed and are prone to crack with cycling. In order to avoid such cracking, yolk shell type coatings were studied.^16^ Sulfur encapsulation using graphene has also been investigated.^17,18^ Similarly, physical trapping of polysulfides has been investigated using carbon nanotube mat electrodes.^19,20^ This allowed the use of a fiber matrix both as an active material for storage and as a barrier for leaking polysulfides. In all the techniques prior to this, cyclability of sulfur electrodes had not been improved considerably, since the physical trapping mechanism did not support long-term encapsulation for dissolved polysulfides. Use of a fiber matrix has improved both electronic conductivity and electrolyte channeling. Nanocomposites of sulfur chemically bonded with carbon were also investigated.^21^ The chemically bonded sulfur however does not seem to participate in lithiation reaction while unbonded sulfur is found to be electrochemically active. Soluble polysulfides tapping by chemisorption of amine-functionalized carbon has been reported in^22^ with a significant capacity retention. In addition to the above modifications of cathode electrodes, the use of solid state electrolytes and gel polymer electrolytes are being investigated to alleviate the sulfur dissolution problem.^23,24^ The development and study of solid or gel electrolytes with high lithium diffusion is a research topic of interest in itself. A good comprehensive review of electrolytes for lithium sulfur batteries can be found in.^25^ There have been some reports on other liquid electrolytes to improve the cyclability of Li–S batteries.^26,27^ Interestingly, the dissolution of polysulfides near the electrode–electrolyte interface is seen as a necessary step for complete lithiation of sulfur, *i.e.*, 2Li + S → Li_2_S. Use of a polymer electrolyte would therefore hinder the intermediate polysulfide formation, lowering the discharge capacity of sulfur. In ref. 28, discharge capacity for the all solid state Li–S battery has been demonstrated as 200 mA h g^−1^ at the 50^th^ cycle which is a considerably lower gravimetric capacity compared to organic electrolyte based Li–S batteries. Due to these reasons, numerous studies were focused on using liquid electrolytes, but trapping lithium polysulfides in porous carbon electrodes such as carbon nanotubes.^29–33^

In our work, a mesoporous titania layer is utilized to trap the dissolved polysulfides along with an unique electrical bridging technique to improve the electrical conductivity within the bulk electrode. The titania particle coating is seen to improve the durability and high capacity retention of the sulfur cathode in two ways: (a) the meso-porosity will enable bulk diffusion of lithium through the pores; and (b) high surface area of titania will promote adsorption of polysulfides. As shown in ref. 34, S^2−^ ions can be adsorbed on titania surfaces. The role of the meso-porous titania coating of the sulfur cathode is thus investigated in order to understand the underlying mechanisms of sulfide ion dissolution and its effect on capacity retention and durability. Sulfur cathodes comprised of titania/carbon have already been investigated by several other groups^35–37^ for Li–S batteries. However, in this work, we have attempted a new approach of fabricating the sulfur electrode by simply coating the sulfur impregnated carbon matrix with meso-porous titania rather than mixing the carbon particles with titania.

## Experimental method

The electrode material is used for coating titania is activated carbon (ACP) derived from bio mass. Sulfur is thermally diffused into the electrode. The synthesis of activated carbon from bio mass is described in the ESI.† The electrode is prepared by ball milling ACP with 10 ml of 60% PVDF (polyvinylidene fluoride) in NMP (*N*-methyl-2-pyrrolidone) solvent for 12 hours. Then the ACP slurry is poured onto a clean glass surface to form a free-standing ACP sheet.

**Fig. 1:**
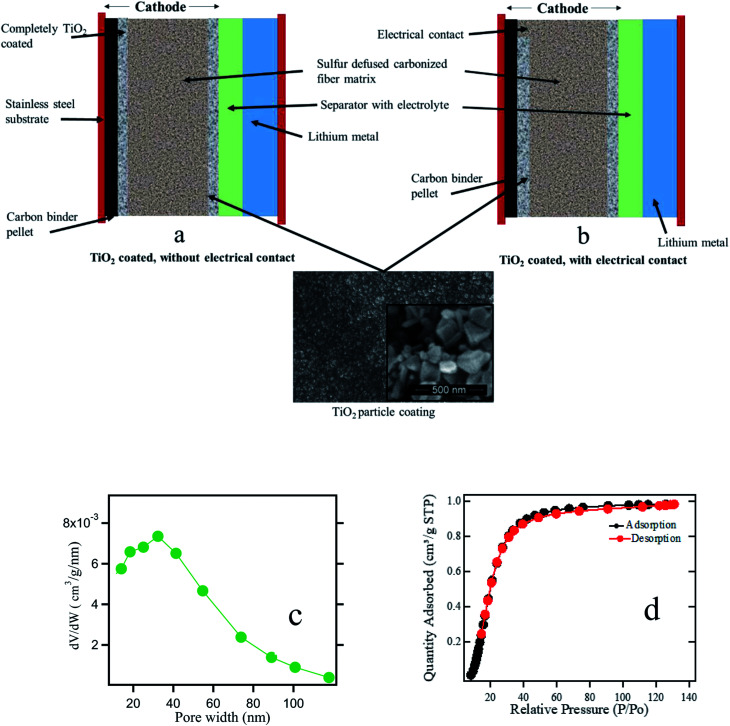
Schematic diagram of the titania coated electrode (a) without electrical contact (b) with electrical contact for ACP supported sulfur cathode in a Li–S cell. The SEM images of the sulfur support and the titania coating and (c) pore width distribution (d) N_2_ adsorption–desorption isotherms for anatase titania powder are also shown.

In the second step, sulfur (3–4 mg) is melted on a hotplate at 130 °C and is impregnated into ACP free standing carbon structures by pressing them onto the melted sulfur. In the third step, ACP electrodes are coated with 200 nm titania paste by dipping the electrodes in titania suspension in ethanol, followed by air drying for 24 hours. A part of the back surface of the titania coating is scratched-off to expose the interior of the carbon/sulfur electrode (bridging) in order to make better electrical contact with the current collector. Then titania coated sulfur electrodes are pressed against a carbon black pellet forming the electrical bridge. The carbon black pellet is made by mixing 20 mg of acetylene carbon black and 20 ml of PTFE (polytetrafluoroethylene). Next, it is placed on a stainless-steel mesh with a diameter of 15 mm and pressed under ∼300 kg of pressure (using a hydraulic press) to mount the entire assembly on the current collector. The thickness of the carbon black pellet is reduced to about 0.1–0.2 mm after pressing. The cathode is then assembled in a CR2032 coin cell with pure lithium metal as the anode, inside an argon-filled glove box. Celgard 3401 polymer separator (∼75 μm thick) is placed between the electrodes. The composition of the electrolyte used in this work is 1 : 1 ratio of 1,2-dimethoxyethane (DME Sigma Aldrich) and 1,3-dioxolane (DOL Sigma Aldrich) in 1 M bis(trifluoromethane)sulfonimide lithium salt (LiTFSI) and 1% wt of LiNO_3_ for a total of 0.5 ml of electrolyte. The ionic conductivity of the electrolyte is ∼14.7 mS cm^−1^ at 25 °C. LiNO_3_ is widely used as an additive in the electrolyte to form a protective film on the lithium anode. Fig. 1 represents the side view of the cell including the SEM image of the titania coating, and the pore width distribution for anatase titania powder measured by BET technique. The mean pore width is found to be 40 nm which is mesoporous. The size of titania particles used in this experiment is around 200 nm. When the electrode is fabricated, we found that the particle coating maintains the meso-porosity.

## Electrochemical and structural analysis

The cells were cycled between 1.5 and 2.8 V *versus* Li/Li^+^ in galvanostatic mode using 16 channel Arbin battery test system. Cyclic voltammetry (CV) was performed at a scan rate of 0.3 V in the range of 1.5 to 2.8 V using the biologic sp-200 electrochemical system. AC impedance (EIS) of the cell was measured using the same electrochemical system over the 1 mHz to 1 MHz range. Both CV and EIS measurements were conducted by a swagelok-based three-electrode configuration with lithium as both the counter electrode and the reference electrode. All performances were carried out at 25 °C.

## Characterization

The ionic conductivity was measured by a biologic sp-200 system. The electrode surface morphology before and after cycling was characterized by a TESCAN thermionic emission scanning electron microscope. X-ray photoelectron spectroscopy (VG scientific-MultiLab 3000) was employed to detect the chemical composition of the cathode. All XPS spectra were fitted with Gaussian–Lorentzian functions and a shirley and linear type background. 2P_3/2_ 2P_1/2_ peaks were fitted using Lorentzian function. The binding energy values were all calibrated using carbon 1S 284.5 eV. Samples for SEM and XPS characterization were prepared by disassembling cells and rinsing with 1,2-dimethoxyethane, 1,3-dioxolane. TGA studies were done by thermogravimetric analyzer TA 2050.

## Results and discussion

**Fig. 2:**
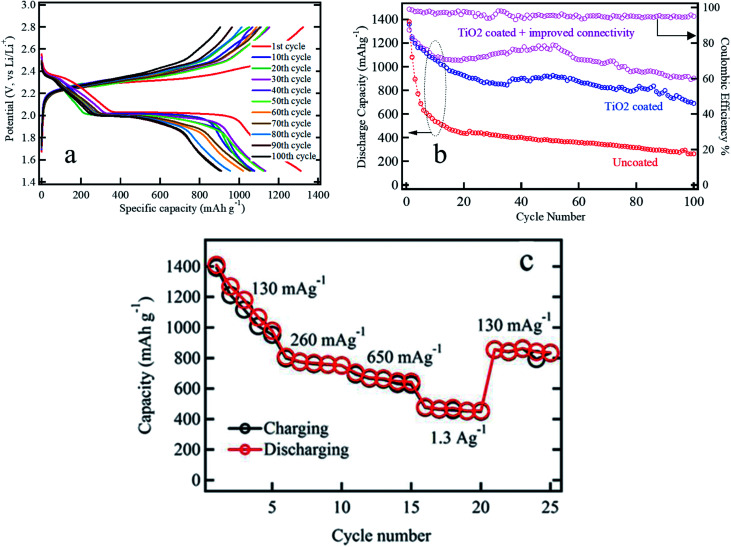
Gravimetric capacity results of ACP based sulfur electrodes; (a) potential *vs.* specific capacity curves of optimized ACP based titania coated sulfur cathode, (b) comparison of the discharge capacity of various ACP based cathodes; (i) titania coated sulfur cathode with (purple) and without (blue) improved electrical connection to the current collector and (ii) uncoated electrodes (red); left axis represent the discharge capacity and the right axis represents the coulombic efficiency for the optimized sulfur cathode (c) rate capability performances of ACP based titania coated sulfur electrode with improved electrical connection.

Performance of the sulfur cathode is tested against lithium metal as the anode in a coin cell configuration over the voltage range of 2.8–1.5 V using an Arbin battery tester. The electrochemical performances of an uncoated sulfur cathode and a mesoporous titania coated cathode at C/3 rate are shown in Fig. 2. The areal sulfur loading is 2.65 mg cm^−2^ and mass of titania coating was approximately 1.5 mg cm^−2^. In all cases of Fig. 2b, discharge curves showed two discharge plateaus at 2.4 and 2.0 V. The sudden drop of voltage in Fig. 2a from 2.6 V to 2.4 V is due to the polarization and *IR* drop of electrodes and electrolyte. The plateau at 2.4 V is believed to be due to the reduction of S_8_ to high-order soluble lithium polysulfides (*e.g.* Li_2_S_4_), and the plateau at 2.0 V is due to further reduction of Li_2_S_4_ into insoluble Li_2_S. The uncoated sulfur electrode shows rapid decay of gravimetric discharge capacity within the first 20 cycles (Fig. 2b). There, titania coated electrode shown in purple color however, exhibited stable discharge capacity in excess of 900 mA h g^−1^ even after the 100^th^ cycle.

The influence of proper electrical connection to the conductive matrix of the electrode is investigated by comparing three different activated carbon electrode systems: (i) uncoated, (ii) titania coated with poor electrical connectivity, and (iii) titania coated with improved electrical connectivity (electrical bridging) as shown in Fig. 2. Experiments were carried out at C/3 discharge and charge rate. Synthesis of activated carbon is described in the ESI.† A properly working sulfur electrode has two voltage plateaus at ∼2.4 V (formation of Li_2_S_*x*_ polysulfides) and ∼2.0 V (formation of Li_2_S and Li_2_S_2_). This is an indication that Li^+^ transport has not been mitigated by the titania particle barrier. A stable discharge capacity of about 980 mA h g^−1^ for 100 cycles has been achieved for the titania coated ACP supported sulfur electrode with improved electrical conductivity. In contrast, the titania coated ACP supported sulfur electrode with poor electrical connectivity shows lower discharge capacity of ∼700 mA h g^−1^ after 100 cycles while the ACP supported sulfur electrode without a coating layer shows discharge capacity of only 265 mA h g^−1^ at 100^th^ cycle. The idea of coating the back side of the electrode with mesoporous titania is to prevent any leak of soluble polysulfides into the electrolytes when the battery is at idle between cycles.

**Fig. 3:**
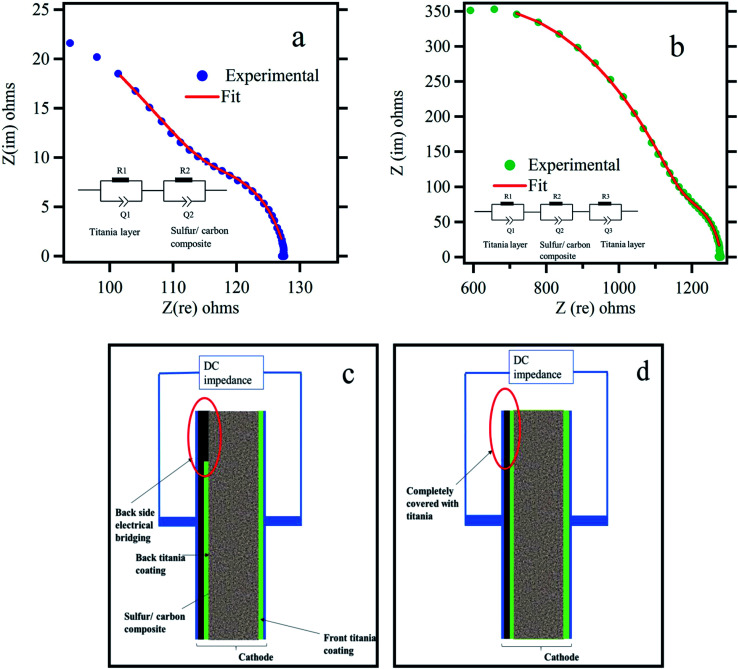
Electrical conductivity measurements of the cathode material: Nyquist plots for titania coated sulfur electrode (a) with part of the backside uncoated (exposed) (b) both sides coated with titania. The insets show the equivalent circuit network utilized for impedance analysis. (c) and (d) show the side view of the titania coated electrode with provisions for electrical bridging and the electrode with both sides completely coated with titania respectively.

Polysulfide trapping by using metal oxide such as titania has been investigated in three different methods by other groups: in the first method, sulfur cathodes were made by simply mixing titania particles with sulfur/carbon composites.^38–40^ In the second method, sulfur was first coated with titania followed by carbonization.^35,41–43^ In the third method, titania nanoparticles have been coated on the polymer separator, forming an effective polysulfide adsorbing barrier.^44^ In all three methods, electrical conductivity between current collector and active material is established only through the carbon matrix in the composite. However, in this work the electrical conductivity between current collector and active material is established through an electrical bridging technique (Fig. 3c). Its effect is further analyzed by 2-probe impedance tests as shown in Fig. 3. The electrical contacts were made to the current collector and the titania coating layer on the other side. It is found that the dc resistance for the electrically bridged cathode is 127.88 Ω in comparison to the dc resistance value of 1283.63 Ω for the electrode with titania coating on both sides. This is a significant improvement in the net electrical resistance due to the electrical bridging leading to a high discharge capacity as seen in Fig. 2b. A complete impedance analysis is presented in Fig. S5 and Table S1,† according to the equivalent circuits proposed in Fig. 3.

In order to investigate high-power performance of the titania coated sulfur electrode with proper electrical contact, rate capability was studied in the voltage range of 2.8–1.5 V with different current densities as shown in Fig. 2c. Five initial formation cycles have been shown at 130 mA g^−1^ current density followed by 5 cycles each at 260 mA g^−1^, 650 mA g^−1^, and 1.3 A g^−1^ current densities. It shows that discharge capacities at 130 mA g^−1^, 260 mA g^−1^, 650 mA g^−1^, and 1.3 A g^−1^ are approximately 1000, 800, 700, and 450 mA h g^−1^, respectively. When the current density is reduced back to 130 mA g^−1^ after the rate performance testing, the sulfur cathode can retain the discharge capacity close to the formerly measured value of 900 mA h g^−1^, indicating its good reversibility and high rate capability and demonstrating the recovery of the titania coated sulfur cathode after subjecting it to different charge–discharge rates.

**Fig. 4:**
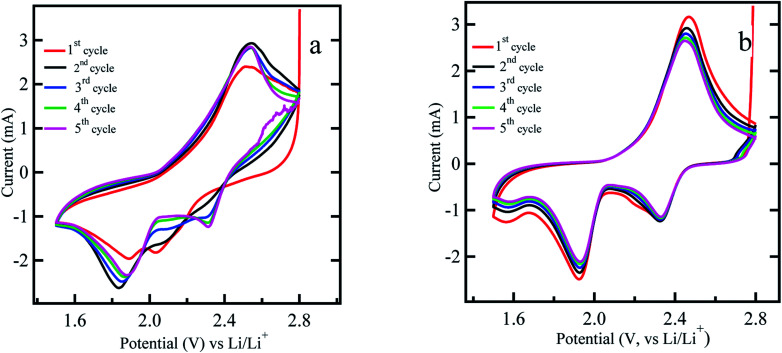
Cyclic voltammetry measurement of (a) uncoated sulfur electrode (b) titania coated sulfur electrode (ACP based) at the voltage range 2.8–1.5 V at scan rate of 0.3 mV s^−1^.

Next, cyclic voltammetry (CV) was carried out for the ACP based sulfur impregnated electrodes without and with titania coating as shown in Fig. 4 within 2.8 V and 1.5 V range at 0.3 mV s^−1^ rate. The lower end potential is chosen to be 1.5 V since LiNO_3_ additive tends to be reduced irreversibly at the voltages below 1.5 V.^45^ CV measurements are carried out for up to 5 cycles and both electrodes showed the complete two step redox reactions with two reduction peaks appearing at around 2.3 and 2.0 V and one oxidation peak at ∼2.4 V. The peak at ∼2.3 V is ascribed to the reduction of sulfur to form the higher order lithium polysulfides (Li_2_S_*n*_, *n* > 4), and the peak at ∼2.0 V corresponds to further reduction of these lithium polysulfides to lower order lithium polysulfides (Li_2_S_*n*_, *n* < 4) including Li_2_S_2_ and Li_2_S. The oxidation peak at ∼2.4 V can be attributed to the oxidation of lithium polysulfides (Li_2_S_*n*_, *n* < 4) back to higher order lithium polysulfides (Li_2_S_*n*_, *n* > 4). Theoretically, two distinct oxidation peaks are expected for the sulfur cathode. However, in our case, the two oxidation peaks appear to merge into a single composite peak. We believe that, the resolution of the oxidation peaks in Li–S battery depend on the charge transfer resistance in the sulfur electrode. Sulfur cathode with better charge transfer properties, will allow all the polysulfide species to oxidize in parallel reactions, result in one convoluted peak. The charge transfer resistance depends on the electronic conductivity, porosity, and the surface area of the conductive material of the sulfur cathode. The work reported in ref. 19 also suggests a similar finding that the oxidation peaks in the cyclic voltammetry curves become more convoluted and less resolved with increased surface area and better charge transfer properties. The CV curve shown in Fig. 4b and discharge curves of Fig. 2b for a titania coated sulfur cathode shows remarkable durability over the cycles. This is an indication of the reformation of sulfur within the bulk electrode and minimal leakage of polysulfides into the electrolyte. In contrast, the uncoated sulfur electrode shows significant irreversibility in the CV diagram with shifting of peak positions and changes in current levels implying dissolution of polysulfides into the electrolyte. In this work, titania is expected to trap the polysulfides and the CV curves should not show a considerable current at the 2.8 V vertex as in Fig. 4b. This reasoning is confirmed by comparing the CV cycles of the uncoated sulfur electrode shown in Fig. 4a. It is noticeable that at 2.8 V vertex of Fig. 4a, there is a cathodic current ∼2 mA, implying existence of dissolved polysulfides in the electrolyte still undergoing oxidation.

**Fig. 5:**
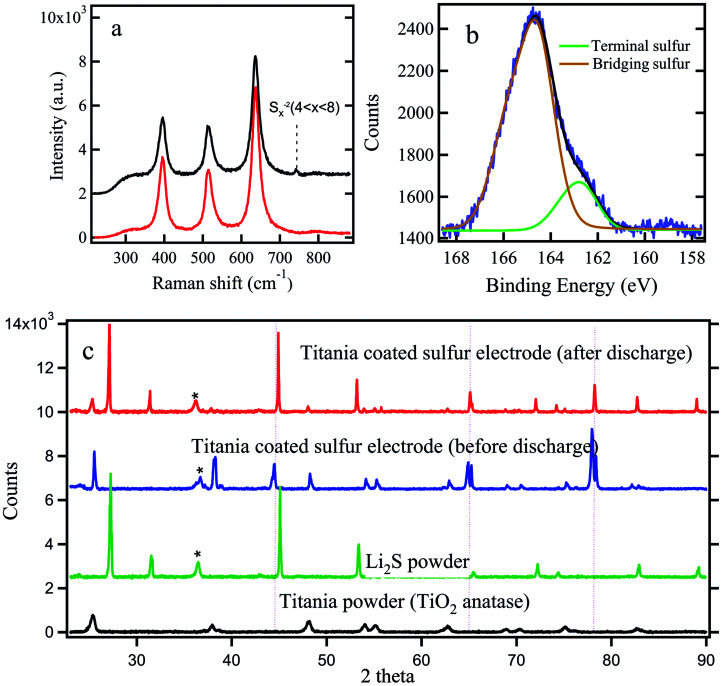
(a) Raman spectra of titania coated sulfur electrodes (ACP based) before and after discharge (b) XPS surface analysis for titania coating (c) XRD spectra of titania coated sulfur electrodes before and after discharge, spectra for Li_2_S and TiO_2_ powders are also shown for comparison. The * represents the signature of the polymer bag and the dotted vertical lines represent the aluminum substrate.

Raman and X-ray photo electron (XPS) analysis were carried out to further confirm the trapping of polysulfides in titania layer. In Raman spectrum analysis, we investigated titania coated electrode before and after discharge as shown in Fig. 5a. Both spectra show 3 clear peaks characteristic of crystalline anatase titania. An additional weak peak appearing at ∼742 cm^−1^ for the discharged electrode can be interpreted as due to the polysulfide links (S_*x*_^2−^, *x* = 4–8).^46^

Fig. 5b shows the sulfur 2P peak (S^2P^) with 2 distinct peaks at 160.4 and 161.9 eV corresponding to bridging sulfur and terminating sulfur respectively.^40^ This is possible due to the efficient trapping of higher order soluble polysulfides in the mesoporous TiO_2_ layer.

The electrode which used in this analysis are washed with the 1 : 1 ratio of 1,2-dimethoxyethane (DME Sigma Aldrich) and 1,3-dioxolane (DOL Sigma Aldrich) to remove any dissolved polysulfide from the surface of titania coating which might not have adhered to the titania surface. Thus, it is reasonable to conclude that the polysulfides detected by Raman and XPS are from the polysulfides which were adhered on titania particles. Fig. 5c compares XRD spectra for titania coated sulfur cathode before and after discharge. XRD spectra for pristine titania and Li_2_S are also shown for comparison. It shows clear evidence of the presence of solid Li_2_S after the first cycle discharge.

**Fig. 6:**
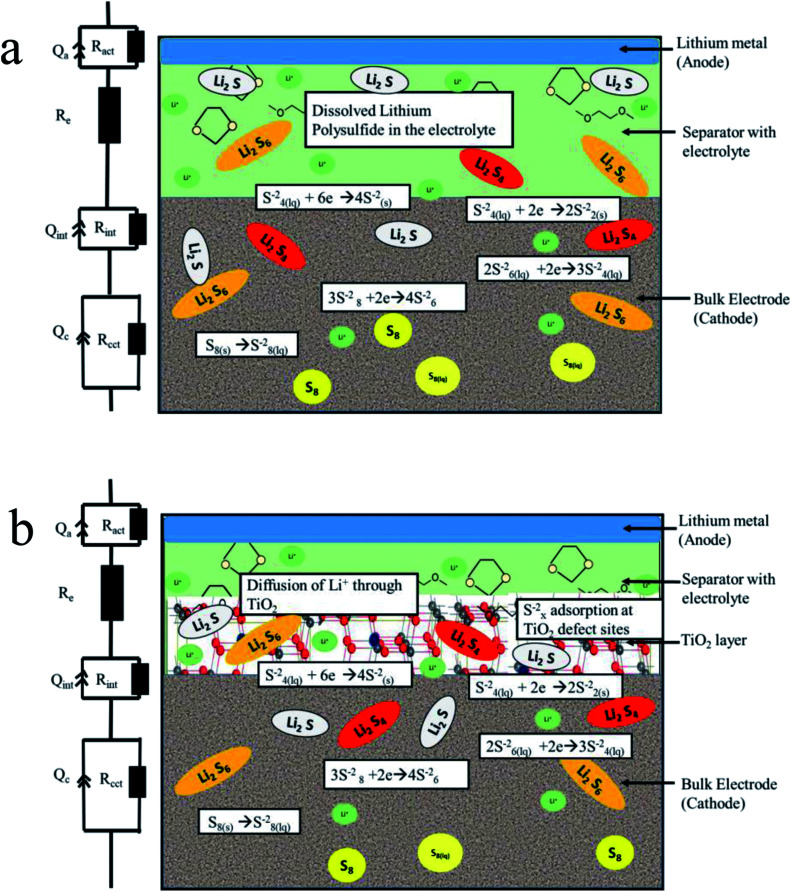
Schematics of the chemical processes in (a) uncoated and (b) titania coated sulfur electrode in Li–S battery.

In the uncoated sulfur electrode, soluble polysulfides are expected to dissolve into the electrolyte. In the case of meso-porous titania coating, the dissolved polysulfide ions adsorb on titania surfaces and never reach bulk electrolyte beyond titania barrier. In the schematic diagram in Fig. 6, the processes of a Li–S battery with uncoated and titania coated cathodes have been categorized into several regions where key reactions take place.

EIS was used to determine impedances within coated and uncoated sulfur cathodes during cycling. Based on the Nyquist plots (Fig. S4a and b in the ESI†) for coated and uncoated sulfur cathodes, an equivalent circuit has been proposed as presented in Fig. 6. Here, *R*_e_ represents the electrolyte resistance as a single series resistance in the network. The loops in the Nyquist plot consisting of superposition of multiple semicircles are each represented by a combination of a resistance and a constant phase element (CPE) in parallel. A similar equivalent circuit modeling and electrochemical impedance analysis can be found in the ESI of the work reported in ref. 47.

**Fig. 7:**
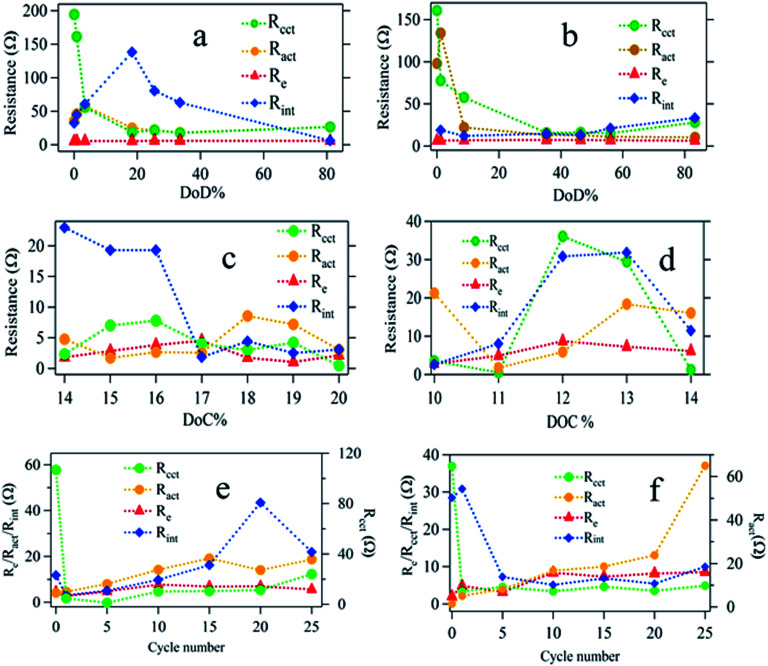
Fitting parameters of EIS data for ACP based sulfur cathode to an equivalent electrical circuit model: each plot contains charge transfer resistance at cathode (*R*_cct_), charge transfer resistance at anode (*R*_act_), electrolyte resistance (*R*_e_), and interface resistance (*R*_int_). Plots (a) and (b) represent results for titania coated and uncoated samples respectively against DOD; plots (c) and (d) represent results for coated and uncoated samples respectively against DOC. Plots (e) and (f) represent results for titania coated and uncoated samples respectively against cycle number.

The choice of a CPE instead of a capacitor is due to the non-ideal behavior of the electrodes. Each semicircle represents (i) charge transfer at the cathode (*R*_cct_‖CPE_cct_) (ii) charge transfer at the anode (*R*_act_‖CPE_act_), and (iii) contact interphase at the cathode (*R*_int_‖CPE_int_) which is present in the bulk of the cathode representing the charge conduction between the cathode current collector and the redox sites in the cathode. The variation of *R*_int_ in the case of titania coating is expected to be significant. The contribution of the anode impedance is neglected because the anode impedance in an electrolyte with polysulfides is small. Fig. 7 shows the relevant impedance parameters extracted by fitting the EIS data with the proposed equivalent circuit during (i) discharging, (ii) charging, and (iii) cycling processes.

The Nyquist plots of the impedance data for uncoated and titania coated sulfur electrode during the discharge are shown in Fig. S4a and b in the ESI.† Only a few selected sets of data are shown for clarity and a typical fitting procedure is shown Fig. S4c† for a selected data set. The discharge curve has been categorized into three zones according to the key actions taking place in the cell. In zone 1, both electrodes are polarized, and solid sulfur starts to dissolve in the electrolyte. In zone 2, longer polysulfide chains are shortened *via* further reduction (in the presence or absence of titania). In zone 3, solid Li_2_S and Li_2_S_2_ are formed. These solid products are more ionic in nature. In Fig. 7a and b, fitting parameters corresponding to charge transfer resistance (*R*_cct_) and interphase resistance (*R*_int_) at cathode for titania coated and uncoated sulfur electrode respectively during discharge are shown at various depth of discharge (DOD).

The behavior of the charge transfer resistance, *R*_cct_ at the cathode is similar in both cases. They both show initial decrease of *R*_cct_ reaching a minimum ∼40% DOD followed by a slow increase. The initial decrease of *R*_cct_ can be interpreted as due to the improved electrochemical accessibility of solid sulfur (insulating) to undergo polysulfide formation. The following increase in *R*_cct_ is due to the formation of insulating and insoluble Li_2_S and Li_2_S_2_. For both coated and uncoated sulfur electrodes this charge transfer process is similar. However, *R*_int_ shows distinctly different behaviors for coated and uncoated cathodes. In the case of titania coated cathodes, *R*_int_ value is seen to increase in zone 1, presumably due to the adsorption of dissolved polysulfides at the defect sites of titania. It is interesting to observe that the interphase resistance drops in zone 2, where longer polysufides are reduced to shorter polysulfides.

During the charging of the cell, solid Li_2_S and Li_2_S_2_ should eventually oxidize back to elemental sulfur through intermediate polysulfide formation. The analysis of the variation of the *R*_int_ during the charging process provides useful information about the underlying mechanism of the titania coated electrode as shown in Fig. 7c. Once Li_2_S starts to oxidize to intermediate polysulfide chains, the interphase resistance, *R*_int_ is expected to decrease as the conductivity improves for the titania coated electrode (Fig. 7c). In contrast, the *R*_int_ of the uncoated sulfur electrode is seen to increase as charging progresses (Fig. 7d). It is reasonable to assume that this conversion (Li_2_S/Li_2_S_2_ to intermediate polysulfides) may take place at the electrode matrix–electrolyte interface since there are considerable amounts of dissolved polysulfides remaining in the electrolyte. As the sulfur growth takes place on the surface, the interphase resistance, *R*_int_ continues to increase (Fig. 7d). It is also noted that, the effect on electrolyte resistance due to the dissolved polysulfide is considerably small (Fig. 7e) in the case of titania coated cathode.

Dissolution of polysulfides increases the viscosity of the electrolyte causing an increase in electrolyte resistance (*R*_e_) noticeable in Fig. 7e and f in different magnitudes. Titania coated sulfur electrodes show a stabilized *R*_e_, however, due to the adsorption activity of titania layer. It is evident from the relative magnitudes of the changes in *R*_e_ that coating of the sulfur electrode with titania has significantly limited the polysulfide dissolution into the electrolyte. Finally, variation of, *R*_act_ during cycling is presented in Fig. 7e and f for both coated and uncoated cathodes as evidence for polysulfide shuttling and Li_2_S and Li_2_S_2_ formation on the anode surface. Li_2_S and Li_2_S_2_ are known to be formed on the anode by reducing the dissolved polysulfides (from cathode) in the electrolyte after shuttling to anode. For the uncoated sulfur cathode, *R*_act_ increases almost linearly until 20^th^ cycle and then shows an abrupt rise confirming the continuous formation of Li_2_S/Li_2_S_2_ on the anode. On the contrary, the titania coated cathode shows saturation of *R*_act_ after the 20^th^ cycle implying limited formation of Li_2_S/Li_2_S_2_ as a result of encapsulation of soluble polysulfide within the titania coating.

## Conclusion

Titania coating of the sulfur electrode with proper electrical contact with the current collector has proven to be effective to enhance the cyclability of Li–S batteries by retaining a stable capacity of 980 mA h g^−1^ discharge profile over 100 cycles. The performance of mesoporous titania coated sulfur was compared with that of uncoated sulfur electrodes using EIS and CV techniques. The mechanism of trapping dissolved polysulfide within the titania layer was verified by investigating *in situ* impedance measurements. *R*_act_ of the cell with titania coated sulfur electrode was stabilized at 20 Ω while *R*_act_ for uncoated sulfur electrode continued to rise beyond 20 Ω during charging and discharging. Such increase in charge transfer resistance at the anode in uncoated sulfur cathode is due to deposition of solid Li_2_S on lithium metal anode. The electrical bridging technique to improve the electrical conductance between the interior of the sulfur/carbon composite and the current collector is proven to contribute significantly for the superior performance of titania coated sulfur electrodes. Otherwise, the role of titania to improve the cyclability of sulfur electrode with high discharge capacity will be undermined due to the poor electrical conductance between the interior of the electrode and the current collector. In addition, Raman and XPS analysis confirm the effective polysulfide trapping by the mesoporous titania coating even though the isolation of different polysulfide species was difficult. Finally, the XRD analysis concludes non-existence of any phase changes in titania confirming that the polysulfide is trapped only by adsorption onto titania.

## Conflicts of interest

The authors declare no competing financial interest.

## Supplementary Material

RA-008-C8RA01380B-s001

## References

[cit1] Manthiram A. (2014). *et al.*, Rechargeable lithium-sulfur batteries. Chem. Rev..

[cit2] Lu J. (2014). *et al.*, Aprotic and aqueous Li-O(2) batteries. Chem. Rev..

[cit3] Zhu J. (2017). *et al.*, A stable organic–inorganic hybrid layer protected lithium metal anode for long-cycle lithium-oxygen batteries. J. Power Sources.

[cit4] Zhang S. S. (2013). Liquid electrolyte lithium/sulfur battery: Fundamental chemistry, problems, and solutions. J. Power Sources.

[cit5] Barchasz C. (2012). *et al.*, Lithium/sulfur cell discharge mechanism: an original approach for intermediate species identification. Anal. Chem..

[cit6] Yin Y. X. (2013). *et al.*, Lithium-sulfur batteries: electrochemistry, materials, and prospects. Angew. Chem., Int. Ed..

[cit7] Mikhaylik Y. V., Akridge J. R. (2004). Polysulfide Shuttle Study in the Li/S Battery System. J. Electrochem. Soc..

[cit8] Xiao J. (2015). *et al.*, Following the transient reactions in lithium-sulfur batteries using an in situ nuclear magnetic resonance technique. Nano Lett..

[cit9] Park J.-W. (2013). *et al.*, Solvent Effect of Room Temperature Ionic Liquids on Electrochemical Reactions in Lithium–Sulfur Batteries. J. Phys. Chem. C.

[cit10] Barchasz C. (2013). *et al.*, Revisiting TEGDME/DIOX Binary Electrolytes for Lithium/Sulfur Batteries: Importance of Solvation Ability and Additives. J. Electrochem. Soc..

[cit11] Jozwiuk A. (2016). *et al.*, The critical role of lithium nitrate in the gas evolution of lithium–sulfur batteries. Energy Environ. Sci..

[cit12] Ma L. (2015). *et al.*, Nanomaterials: Science and applications in the lithium–sulfur battery. Nano Today.

[cit13] Li X. (2011). *et al.*, Optimization of mesoporous carbon structures for lithium–sulfur battery applications. J. Mater. Chem..

[cit14] Chen S.-R. (2011). *et al.*, Ordered mesoporous carbon/sulfur nanocomposite of high performances as cathode for lithium–sulfur battery. Electrochim. Acta.

[cit15] Weiyang L., Guangyuan Z., Yang Y., Wei Seh Z., Liu N., Cui Y. (2013). High performance hollow sulfur nanostructured battery cathode through a scalable room temperature one step bottom up approach. Proc. Natl. Acad. Sci. U. S. A..

[cit16] Zhou W. (2013). *et al.*, Yolk-shell structure of polyaniline-coated sulfur for lithium-sulfur batteries. J. Am. Chem. Soc..

[cit17] Xiao M. (2013). *et al.*, Sulfur@graphene oxide core–shell particles as a rechargeable lithium–sulfur battery cathode material with high cycling stability and capacity. RSC Adv..

[cit18] Dai H. (2011). Graphene-Wrapped Sulfur Particles as a Rechargeable Lithium Sulfur Battery Cathode Material with High Capacity and Cycling Stability. Nano Lett..

[cit19] Su Y. S., Fu Y., Manthiram A. (2012). Self-weaving sulfur-carbon composite cathodes for high rate lithium-sulfur batteries. Phys. Chem. Chem. Phys..

[cit20] Singhal R. (2015). *et al.*, A free-standing carbon nanofiber interlayer for high-performance lithium–sulfur batteries. J. Mater. Chem. A.

[cit21] Wang J. L., Yang J., Xie J., Xu N. (2002). A novel conductive Polymer-Sulfur composite cathode material for rechargeable Lithium batteries. Adv. Mater..

[cit22] Ma L. (2016). *et al.*, Enhanced Li-S Batteries Using Amine-Functionalized Carbon Nanotubes in the Cathode. ACS Nano.

[cit23] Zhang S. S., Tran D. T. (2013). How a gel polymer electrolyte affects performance of lithium/sulfur batteries. Electrochim. Acta.

[cit24] Tatsumisago M., Nagao M., Hayashi A. (2013). Recent development of sulfide solid electrolytes and interfacial modification for all-solid-state rechargeable lithium batteries. Journal of Asian Ceramic Societies.

[cit25] Scheers J., Fantini S., Johansson P. (2014). A review of electrolytes for lithium–sulphur batteries. J. Power Sources.

[cit26] Carbone L. (2015). *et al.*, Comparative Study of Ether-Based Electrolytes for Application in Lithium-Sulfur Battery. ACS Appl. Mater. Interfaces.

[cit27] Agostini M. (2015). *et al.*, Polysulfide-containing Glyme-based Electrolytes for Lithium Sulfur Battery. Chem. Mater..

[cit28] Zhu X. (2005). *et al.*, Electrochemical characterization and performance improvement of lithium/sulfur polymer batteries. J. Power Sources.

[cit29] Xu T. (2013). *et al.*, Mesoporous carbon-carbon nanotube-sulfur composite microspheres for high-areal-capacity lithium-sulfur battery cathodes. ACS Appl. Mater. Interfaces.

[cit30] Sun L. (2014). *et al.*, Sulfur nanocrystals confined in carbon nanotube network as a binder-free electrode for high-performance lithium sulfur batteries. Nano Lett..

[cit31] He G. (2013). *et al.*Tailoring porosity in carbon nanospheres for lithium sulfur battery cathodes. ACS Nano.

[cit32] Ding B. (2015). *et al.*, Nanospace-confinement copolymerization strategy for encapsulating polymeric sulfur into porous carbon for lithium-sulfur batteries. ACS Appl. Mater. Interfaces.

[cit33] Song M. K., Zhang Y., Cairns E. J. (2013). A long-life, high-rate lithium/sulfur cell: a multifaceted approach to enhancing cell performance. Nano Lett..

[cit34] Diebold U. (2001). Understanding metal oxide surfaces at the atomic scale: STM investigations of bulk-defect dependent surface processes. Mater. Res. Soc. Symp. Proc..

[cit35] Xu G. (2015). *et al.*, Absorption mechanism of carbon-nanotube paper-titanium dioxide as a multifunctional barrier material for lithium-sulfur batteries. Nano Res..

[cit36] Evers S., Yim T., Nazar L. F. (2012). Understanding the Nature of Absorption/Adsorption in Nanoporous Polysulfide Sorbents for the Li–S Battery. J. Phys. Chem. C.

[cit37] Liang G. (2016). *et al.*, Ultrafine TiO2 Decorated Carbon Nanofibers as Multifunctional Interlayer for High-Performance Lithium-Sulfur Battery. ACS Appl. Mater. Interfaces.

[cit38] Scott Evers T. Y., Nazar L. F. (2012). Understanding the Nature of Absorption/Adsorption in Nanoporous Polysulfide Sorbents for the Li–S Battery. J. Phys. Chem. C.

[cit39] Yang Z. Z. (2016). *et al.*, Hierarchical TiO_2_ spheres as highly efficient polysulfide host for lithium-sulfur batteries. Sci. Rep..

[cit40] Yang X. (2016). *et al.*, Mesoporous TiO_2_ nanosheet with a large amount of exposed {001} facets as sulfur host for high-performance lithium–sulfur batteries. J. Solid State Electrochem..

[cit41] Wei Seh Z. (2013). *et al.*, Sulphur-TiO_2_ yolk-shell nanoarchitecture with internal void space for long-cycle lithium-sulphur batteries. Nat. Commun..

[cit42] Xiao Z. (2015). *et al.*, A Lightweight TiO(2)/Graphene Interlayer, Applied as a Highly Effective Polysulfide Absorbent for Fast, Long-Life Lithium-Sulfur Batteries. Adv. Mater..

[cit43] Wang H. (2014). *et al.*, TiO_2_ coated three-dimensional hierarchically ordered porous sulfur electrode for the lithium/sulfur rechargeable batteries. Energy.

[cit44] Kim M. S. (2016). *et al.*, Multifunctional Separator Coatings for High-Performance Lithium-Sulfur Batteries. Adv. Mater. Interfaces.

[cit45] Zhang S. S. (2012). Role of LiNO_3_ in rechargeable lithium/sulfur battery. Electrochim. Acta.

[cit46] Wu H. L., Huff L. A., Gewirth A. A. (2015). In situ Raman spectroscopy of sulfur speciation in lithium-sulfur batteries. ACS Appl. Mater. Interfaces.

[cit47] Ma L. (2014). *et al.*, Tethered Molecular Sorbents: Enabling Metal-Sulfur Battery Cathodes. Adv. Energy Mater..

