# Supplementary material for: Mesoporous TiO2 coating on carbon–sulfur cathode for high capacity Li–sulfur battery
Source: RSC Adv. 2018 Mar 26;8(21):11622–32. doi: 10.1039/c8ra01380b (PMC9079043; doi:10.1039/c8ra01380b)
Supplement: RA-008-C8RA01380B-s001 [file RA-008-C8RA01380B-s001.pdf]

Electronic Supplementary Material (ESI) for RSC Advances.
This journal is © The Royal Society of Chemistry 2018

Mesoporous TiO_2 coating on carbon-sulfur cathode for high capacity Li-Sulfur battery

Ruchira Dharmasena[a,b], *Arjun Kumar Thapa*[a], *Ram Krishna Hona*[c], *Jacek Jasinski*[a], *Mahendra Sunkara*[a*], *Gamini U. Sumanasekera*[a,b*]

[a]Conn Center for Renewable Energy Research, University of Louisville, KY,USA

[b]Department of Physics & Astronomy, University of Louisville, KY, USA

[c]Department of Chemistry, University of Louisville, KY, USA

[*]Corresponding author: gamini.sumanasekera@louisville.edu or mahendra@louisville.edu

SUPPORTING ONLINE MATERIAL

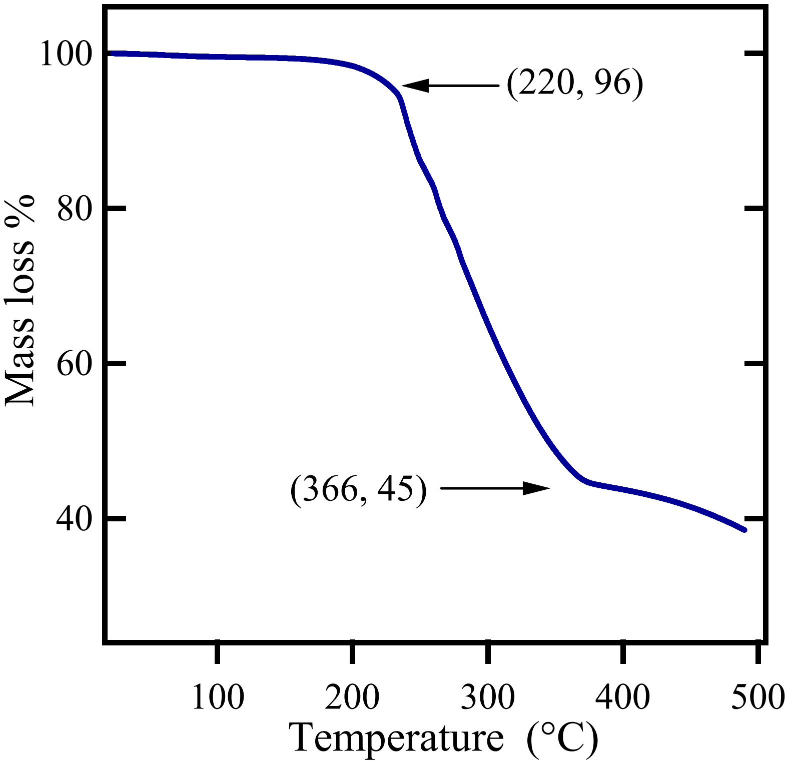

Figure S1: Thermogravimetric analysis (TGA) of sulfur diffused in ACP. Sulfur amount is estimated to be ~2.26 mg.

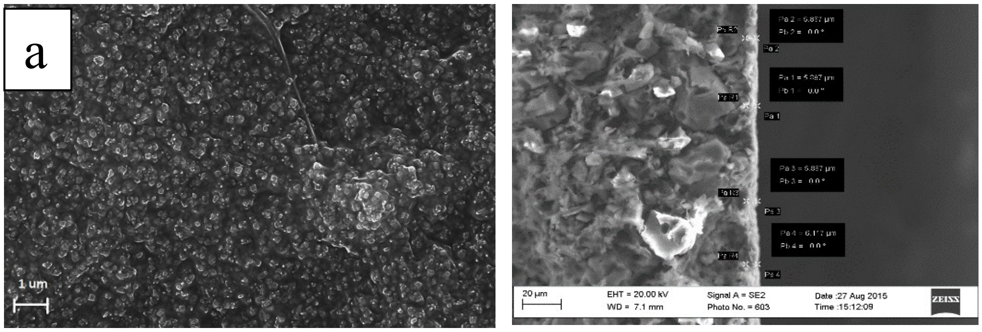

Figure S2: SEM images of (a) Mesoporous titania layer on ACP based sulfur matrix (b) cross sectional view of the electrode showing the thickness of the titania coating layer on ACP sulfur electrode. titania layer has an average thickness of 5.8-6.1 μm.

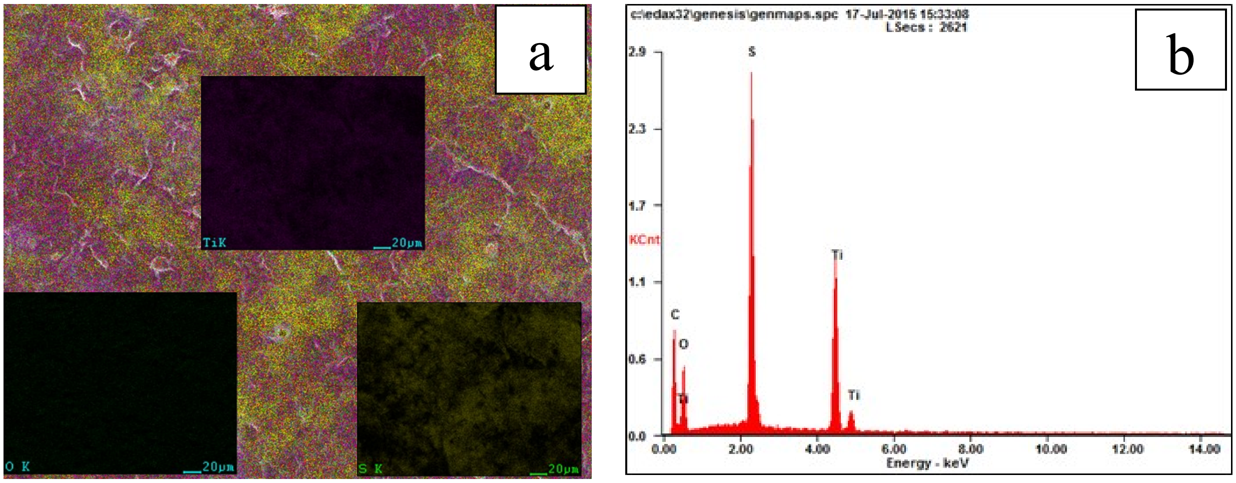

Figure S3: (a) EDAX mapping of the titania coated sulfur electrode; insets show the mapping of Oxygen O K distribution in green, Sulfur S K distribution in yellow, and titanium Ti K distribution in purple (b) EDAX spectrum

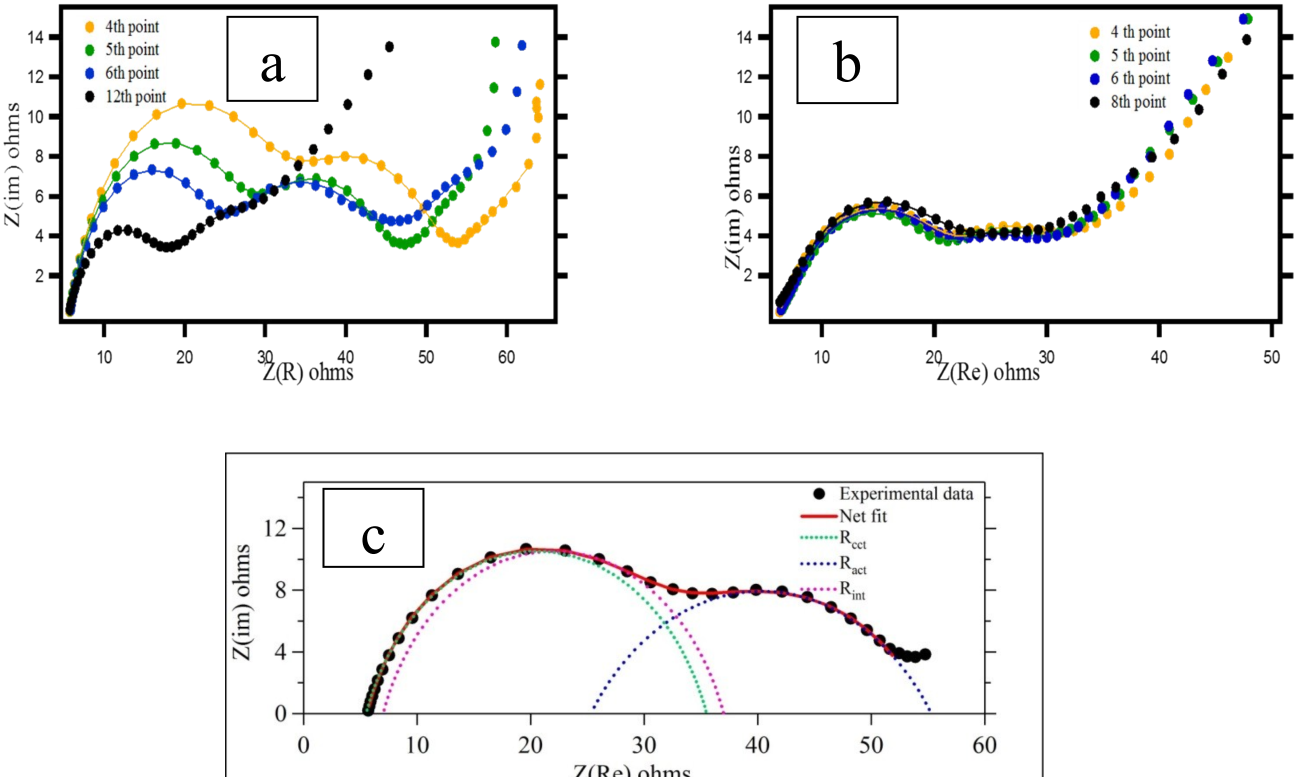

Figure S4: EIS analysis in zone 2 at discharge (a) titania coated sulfur electrode (b) sulfur coated electrode (c) Best fit of the EIS data to the proposed network model.

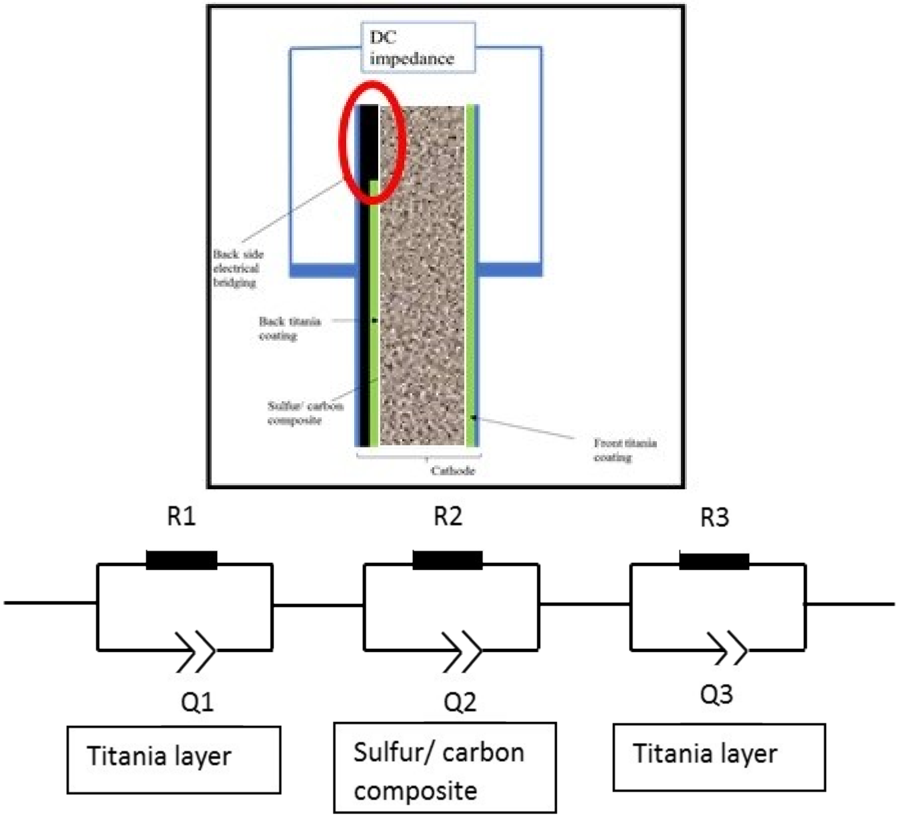

Figure S5: Equivalent circuit model used for fitting the Nyquist plots mentioned in figure 3

Electrode	Resistance (R1)	Resistance (R2)	Resistance (R3)
TiO_2 coated sulfur electrode with back side electrical bridging	119.10 Ω	8.78 Ω	
TiO_2 coated sulfur electrode with both side completely coated	643 Ω	53.63 Ω	587 Ω

Table S1: Fitted resistance values of 2-probe conductivity measurements

Synthesis of activated Carbon (ACP)

Soy hull is used as the source of initial material for synthesizing activated carbon. Potassium hydroxide (KOH) solution is made by dissolving 10 g of 85 % wt KOH pellets in 100 ml of distilled water. Crushed soy hull is mixed with KOH solution at a ratio of 1:1 by mass. The mixture is stirred until it becomes a thick paste. Then, it is allowed to dry at room temperature in a fume hood. The resulting product is then carbonized in a horizontal tube furnace under nitrogen at a positive pressure of .02 MPa at 950 ˚C for one hour. The ramp rate is 10 ˚C/min until 500 ˚C and then is increased to 15 ˚C/min up to the final temperature of 950 ˚C. The sample is then allowed to cool naturally before removing it from the furnace. The samples were washed thoroughly with 100 ml deionized water and dried to remove any excess ions that could potentially block channels.
